# Dexmedetomidine reduces the incidence of postoperative delirium after cardiac surgery: a meta-analysis of randomized controlled trials

**DOI:** 10.1186/s12871-021-01370-1

**Published:** 2021-05-18

**Authors:** Peng Li, Lu-xi Li, Zhen-zhen Zhao, Jian Xie, Cheng-long Zhu, Xiao-ming Deng, Jia-feng Wang

**Affiliations:** Faculty of Anesthesiology, Changhai Hospital, Naval Medical University, 168 Changhai Road, Shanghai, China

**Keywords:** Dexmedetomidine, Postoperative delirium, Cardiac surgery

## Abstract

**Background:**

The role of dexmedetomidine in preventing postoperative delirium (POD) after cardiac surgery remains controversial because of several recent trials with negative results. We aimed to perform an updated meta-analysis of randomized controlled trials (RCTs) to clarify this controversy.

**Methods:**

RCTs investigating the perioperative administration of dexmedetomidine in cardiac surgery were retrieved from PubMed, Web of Science, and the Cochrane library until August,27,2020. Two researchers independently screened the literature, collected the data and evaluated the bias risk of the included studies. The meta-analysis was performed with the RevMan 5.3.

**Results:**

A total of 15 studies including 2813 patients were included in the study. A pooled result showed that dexmedetomidine could reduce the risk of POD in adult population underwent cardiac surgery (OR 0.56, 95%CI 0.36–0.89, *P* = 0.0004, I^2^ = 64%). The subgroup analysis demonstrated that the protective effect of dexmedetomidine was only present in the patients injected with dexmedetomidine after surgery but not from the start of surgery, in the adult patients without specific age limitation but not in the elderly, and in the studies in comparison with other sedatives but not with placebo. There were no statistical differences when analyzing the secondary outcomes including hypotension (OR 1.13; 95% CI 0.54–2.37, *P* < 0.00001, I^2^ = 85%), bradycardia (OR 1.72; 95% CI 0.84–3.53, *P* = 0.04, I^2^ = 58%) and atrial fibrillation (OR 0.87; 95% CI 0.70–1.08, *P* = 0.43, I^2^ = 0).

**Conclusions:**

Dexmedetomidine can reduce the incidence of POD compared to other sedatives and opioids after cardiac surgery in adult patients. The proper population and timing for perioperative use of dexmedetomidine after cardiac surgery remain to be further investigated.

**Supplementary Information:**

The online version contains supplementary material available at 10.1186/s12871-021-01370-1.

## Background

Delirium is an acute brain disorder that involves changes in consciousness, attention, cognition, and perception [1, 2].The incidence of postoperative delirium (POD) is high among patients undergoing cardiac surgery, ranging from 20 to 50%, and the risk is even higher in the elderly [3]. POD may lead to undesirable outcome for the patients and their families, and is associated with increasing nursing home admission, elevated healthcare costs, high morbidity and mortality [4, 5].

Although the risk factors and consequences of POD are well recognized, no pharmacologic agent has been approved to treat this disorder [6]. Several recent meta-analyses of randomized clinical trials have found that dexmedetomidine reduces the incidence of POD in patients after cardiac surgery [7, 8]. However, several recent well-designed large-scale randomized controlled trials failed to find a beneficial effect of dexmedetomidine in preventing POD after cardiac surgery [9, 10]. These studies provided new doubt against previous results of the meta-analyses for perioperative use of dexmedetomidine after cardiac surgery. Therefore, we performed an updated meta-analysis of randomized controlled trials to explore the pooled effects of dexmedetomidine in patients undergoing cardiac surgery with inclusion of the recent trials with negative results.

## Methods

### Protocol and registration

This meta-analysis was conducted in accordance with the recommendations of Preferred Reporting Items for Systematic Reviews and Meta-Analyses (PRISMA) (Supplementary Table. 1) [11].

### Search strategy

Relevant researches published from inception of databases until August 27,2020 were systematically searched by the following databases: Pubmed/Medline, the Cochrane Library/Central, Embase and Web of science. The references included in the study were also reviewed. According to the search strategy, both MeSH terms and free terms were used. A basic search strategy was conducted using the following terms: (dexmedetomidine OR “dexmedetomidine” [MeSH]) AND (“cardiac surgery” OR “heart surgery” OR valve OR CPB OR “cardiopulmonary bypass” OR CAB OR “coronary artery bypass” OR “aortic surgery” OR “congenital heart disease”) in All Fields. Then those studies were screened for POD in the outcome.

### Inclusion and exclusion criteria

Inclusion criteria for this study were as follows: 1) each study contained two comparison groups; 2) one received dexmedetomidine, and the other group received normal saline (NS) or other anesthetic drugs; 3) POD should be included in the primary or secondary outcome; 4) pre-existing cognitive impairment should be excluded by assessment with mini-mental state examination (MMSE) score or other tools; 5) only randomized controlled trials and those published in English language were included to ensure the quality of pooled results [7, 12].

The exclusion criteria included: 1) pediatric surgery; 2) non-cardiac surgery; 3) control group received benzodiazepines; 4) non-intravenous administration of dexmedetomidine; 5) retrospective study, observational study, reviews or animal studies. The articles that failed to provide sufficient information or data were also excluded [7, 12].

### Endpoints

The primary endpoint of this meta-analysis was the incidence of POD after cardiac surgery at any time during a patient’s hospital stay (between ICU admission and hospital discharge, mostly followed up at the 3rd day, 5th day or 7th day), and POD was measured by the Confusion Assessment Method (CAM), CAM-ICU or Richmond Agitation Sedation Scale (RASS). The secondary outcomes included the incidence of bradycardia, hypotension and atrial fibrillation in ICU.

### Data extraction

Data extraction and quality assessment were completed by 2 authors independently and all the information were summarized into a table. The differences in data extraction and quality assessment were discussed and subjected to a third reviewer if no agreement was obtained. The extracted date and information included first author, published year, surgery type, the duration of cardiopulmonary bypass (CPB), timing and dose of dexmedetomidine and the control group and methods of delirium assessment methods. The adverse events including bradycardia hypotension and atrial fibrillation were extracted as well.

### Quality assessment

Quality assessment of included RCTs was performed according to the Cochrane bias risk assessment tool. The criteria were composed of the randomization method, allocation concealment blind of researchers and subjects, blinding to the outcome assessment, incomplete outcome reporting, selective reporting and others.

### Statistical analysis

Meta-analysis was performed using the RevMan 5.3 software to present the dichotomous data (incidence of POD, bradycardia, hypotension and atrial fibrillation). The odds ratio (OR) with a 95% confidence interval (CI) was used to represent the effects of intervention over that of control. The heterogeneity test was evaluated by the I^2^ coefficient. An I^2^ > 75% suggested an obvious heterogeneity between the studies; if the I^2^ < 40%, the study could be considered homogeneous; if the I^2^ was between 30 to 60%, a moderate heterogeneity was considered. According to the results of the heterogeneity calculation, the random effect model was used if there was significant heterogeneity; otherwise, the fixed effect model was used. When significant heterogeneity was indicated, the subgroup analysis of the target data was introduced. The results of meta-analysis were presented in forms of forest plots. And the funnel plot was used to detect publication bias. All significance testing was two-sided, and *P* < 0.05 was considered as statistically significant.

## Results

### Search results and characteristics of included studies

**Fig. 1:**
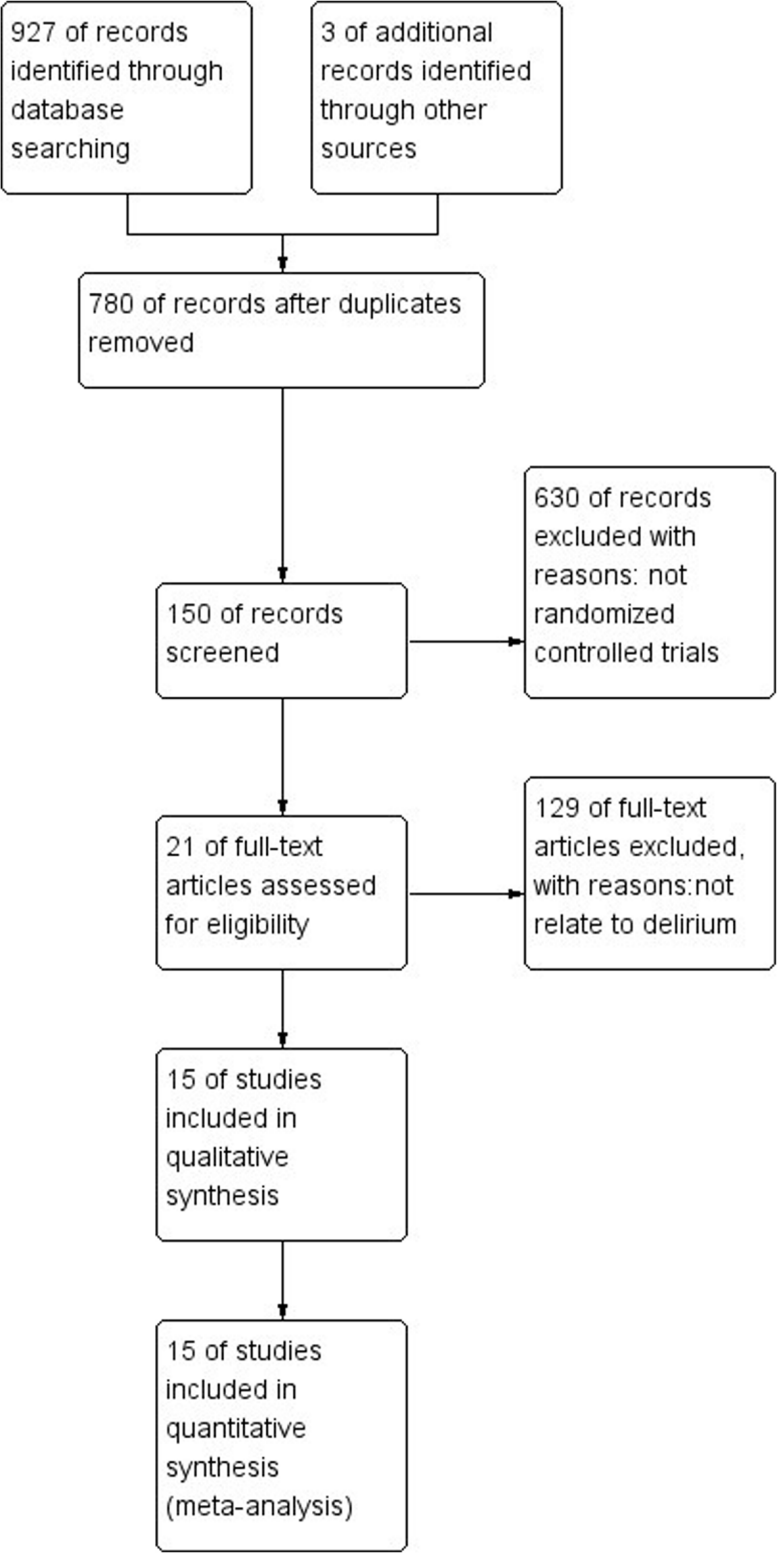
Flow diagram following the PRISMA guideline

**Fig. 2:**
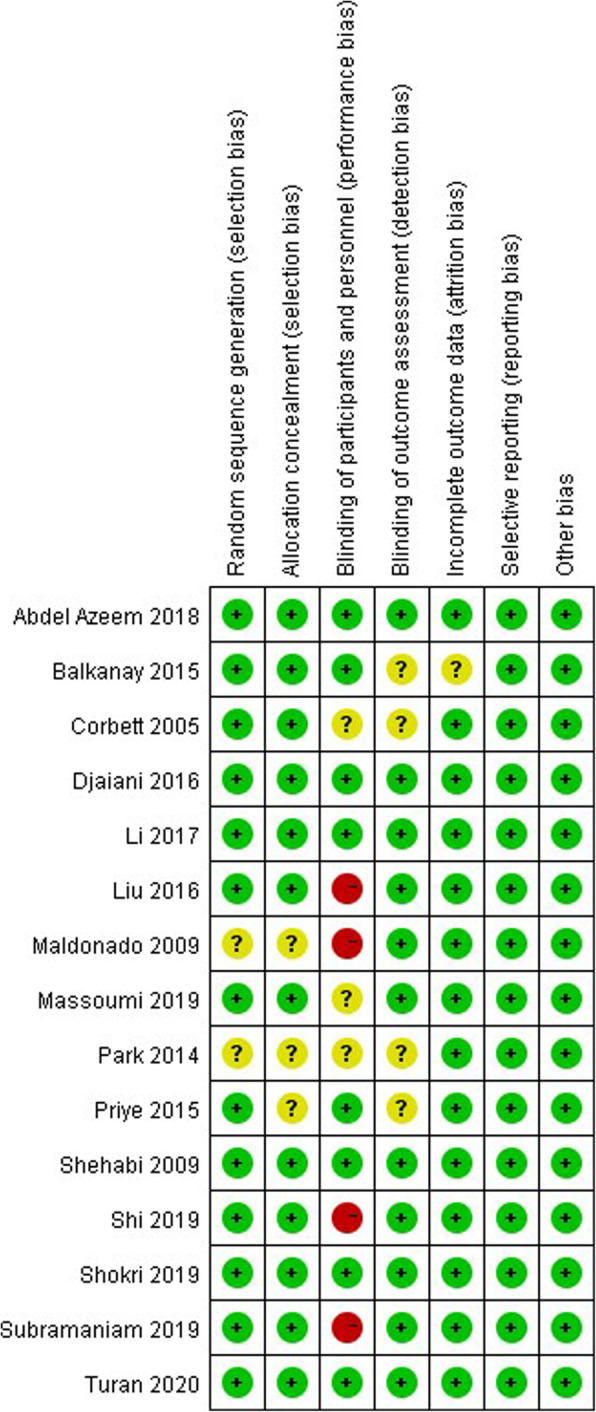
The risk of bias assessment of each included trial according to the Cochrane Risk of Bias Methods

According to the search strategy, a total of 930 trials were retrieved. Among them, 780 studies were removed because of non-randomized design. The other 150 studies were screened strictly based on the inclusion and exclusion criteria, and 129 of them were discarded due to no relevance to POD. Finally, 15 RCTs including 2813 patients were ultimately included in this meta-analysis. The flow diagram following the PRISMA guideline was shown in Fig. 1**.** All the included articles were evaluated with the quality assessment items of Cochrane Risk of Bias Methods. Four trials were evaluated as high risk of performance bias and the others were defined as good qualities (Fig. 2). The major characters of these included studies were extracted and presented in Table 1. There were 1405 patients receiving dexmedetomidine and 1408 receiving control. The control included normal saline in 5 studies [9, 13–16], propofol in 5 studies [4, 10, 17–19], opioid in 4 studies [20–22], and midazolam combined with propofol in 1 study [23].

**Table 1:** The major characters of these included studies

Studies	Publish year	sample size (n)	Age (yr)	Type of surgery	Dexmedetomidine doses	Control	Delirium assessment methods	Time of CPB (min)	Primary Outcomes	Secondary Outcomes
Azeem	2018	60	DEX: 65.3 ± 4.8 CON: 66.7 ± 5.6	MIX	infusion 0.4–0.7 μg/kg/h postoperatively	MOR	CAM-ICU	ND	POD incidence	HR, SBP, DBP, Duration of intubation and mechanical ventilation
Balkanay	2015	88	Mean age: 60.5 ± 8.6	CABG	High-dose DEX group: 0.04–0.50 mg/kg/h Low-dose DEX group: 0.04–0.50 mg/kg/h	NS	ND	NS: 91.1 ± 32.1 DEX1: 98.6 ± 34.7 DEX2: 100.7 ± 29.5	Renal function	POD incidence, length of ICU stay, hospital stay, bradycardia, hypotension, AF, postoperative agitation, furosemide need
Corbett	2005	89	DEX: 63.6 ± 10.1 PRO: 62.4 ± 10.7	CABG	1 mg/kg loading dose, then 0.4 mg/kg/h during MV	PRO	Modified Hewitt questionnaire	ND	Patients’ satisfaction	POD incidence, length of ICU stay, hospital stay, MV, in-hospital mortality, pain, etc
Djaiani	2016	183	DEX: 72.7 ± 6.4 PRO: 72.4 ± 6.2	MIX	0.4 μg/kg infusion over a period of 10-20 min followed by 0.2–0.7 μg/kg/h infusion	PRO	CAM and CAM-ICU	DEX: 100 (71–127) PRO: 98 (77.5–133)	POD incidence	POD duration, length of ICU stay, hospital stay, time to extubation, in-hospital mortality, AF, etc
Li	2017	285	DEX: 66.4 ± 5.4 NS: 67.5 ± 5.3	MIX	0.6 μg/kg infusion for 10 min, then 0.4 μg/kg/h until the end of surgery, 0.1 μg/kg/h after surgery	NS	CAM and CAM-ICU MMSE	DEX: 105 (84 to 129) NS: 101 (81 to 130)	POD incidence	POD duration, length of ICU stay, hospital stay, MV, 30-day mortality, bradycardia, etc
Liu	2016	61	DEX: 53 (48–63) PRO: 55 (48–62)	Cardiac valve surgery	0.2–1.5 mg/kg/h after arrival on ICU before extubation	PRO	CAM-ICU	DEX: 73 (60–88) PRO: 68 (54–80)	microcirculatory variables and clinical parameters	POD incidence, bradycardia, hypotension, nausea, vomiting, AF
Maldonado	2009	90	DEX: 55 ± 16 PRO: 58 ± 18 MID: 60 ± 16	Cardiac valve surgery	Loading dose: 0.4μg/kg, followed by a maintenance drip of 0.2 μg/kg/h– 0.7μg/kg/h	PRO and MID	Diagnostic and Statistical Manual of Mental Disorders	DEX: 165 ± 62 PRO: 162 ± 57 MID: 163 ± 51	POD incidence	Length of stay in ICU and hospital, use of postoperative rescue medications
Massoumi	2019	88	DEX: 61.80 ± 7.90 NS: 61.30 ± 8.90	CABG	1 μg/kg subcutaneously treated within 10 min and 0.2–0.7 μg/kg/h in hour infusion by the syringe pum	NS	RASS CAM-ICU	ND	POD incidence	laboratory variables and vital signs
Park	2014	142	DEX: 51.09 ± 16.10 REM: 54.35 ± 13.97	MIX	0.5 mg/kg loading dose after arrival on ICU, then 0.2–0.8 mg/kg/h until discharged from ICU	REM	CAM-ICU	DEX: 159.55 ± 56.55 REM: 173.19 ± 79.56	POD incidence	POD duration, length of ICU stay, hospital stay, time to extubation; bradycardia,etc
Priye	2015	64	DEX: 45.1 ± 14.7 NS: 41.4 ± 11.9	MIX	0.4 mg/kg/h arrival on ICU for 12 h	NS	RASS	DEX: 98.22 ± 30.61 NS: 95.00 ± 35.51	VAS scores	POD incidence, awakening time
Shehabi	2009	299	DEX: 71.5 (66 to 76) MOR: 71.0 (65 to 75)	MIX	0.1–0.7 mg/kg/h until removal of chest drain	MOR	CAM-ICU	DEX: 98 (80 to 128) MOR: 100 (77 to 120)	POD incidence	Length of stay in ICU and hospital, time to extubation, in-hospital mortality, bradycardia, hypotension, tachycardia, AF, VF, etc
Shi	2019	164	DEX: 74.7 ± 7.2 PRO: 74.2 ± 7.7	MIX	0.4–0.6 μg/kg/h maintenance syringe pump intravenous infusion during operation	PRO	CAM	DEX:110.8 (25.2) PRO: 115.1 (28.9)	POD incidence	POD onset, POD duration, length of stay in ICU and in Hospital
Shokri	2019	286	DEX: 63.75 ± 3.29 CLO: 64.38 ± 4.81	CABG	0.7–1.2 μg/kg/h with an increment of 0.1–0.2 μg/kg/h every 30 min, up to 1–1.4 μg/kg body-weight/h	clonidine	CAM-ICU	ND	POD incidence	Extubation time, lengths of intensive care unit (ICU) and hospital stay, need for inotropic support or vasopressors
Subramaniam	2019	120	DEX: 64 (63–72) and 79 (63–74) PRO: 70 (66–75) and 71 (64–79)	MIX	0.5–1 μg/kg during chest closure 0.1–1.4 μg/kg per h infusion continued to 6 h	PRO	CAM or CAM-ICU	ND	POD incidence	Duration of POD, postoperative cognition at discharge, 48-h break-through analgesic requirements, and ICU and hospital lengths of stay
Turan	2020	794	DEX: 63 ± 11 NS: 62 ± 12	MIX	0·1 μg/kg per h before the surgical incision 0·2 μg/kg per h at the end of bypass, 0·4 μg/kg per h maintained until 24 h postoperatively	NS	RASS CAM-ICU	ND	POD incidence Atrial arrhythmia	Kidney function; 90-daypersistent incisional pain

*DEX* dexmedetomidine, *NS* normal saline, *REM* remifentanil, *MOR* morphine, *PRO* propofol, *MID* midazolam, *POD* postoperative delirium, *CAM* Confusion Assessment Method, *CAM-ICU* CAM for Intensive Care Unit, *RASS* Richmond Agitation Sedation Scale, *MMSE* Mini-Mental State Examination, *MV* mechanical ventilation, *CPB* cardiopulmonary bypass, *CABG* coronary artery bypass grafting, *DEX1* Total dose of Dex < 8 μg/kg group, *DEX2* Total dose of Dex ≥8 μg/kg group, *MIX* Mixed cardiac surgery includes CABG surgery plus valve replacement, CABG surgery plus maze procedure, CABG surgery plus ascending aorta replacement, CABG surgery plus pulmonary vein isolation with or without valve replacement and others, *AF* atrial fibrillation, *ND* No Data

The timing of dexmedetomidine was classified into perioperative use and postoperative use according to the start time of administration. The timing was started from before or after anesthesia induction in the operating room in 5 studies [9, 15, 17, 19, 23] and started chest closure maintained until the end of mechanical ventilation or continued for 24 h in ICU in the rest of included studie s[4, 10, 13, 14, 16, 18, 20–22, 24]..

The surgery type included coronary artery bypass grafting (CABG) in 4 trials [14, 16, 19, 21],valve surgery in 2 trials [18, 23] and mix surgery (CABG plus valve surgery and others) in 9 studies [4, 9, 10, 13, 15, 17, 20, 22, 24]. POD incidence was set as the primary outcomes in most of the studies [4, 9, 10, 14, 15, 17, 20–24], while in 4 trials it was chosen as the secondary outcomes [13, 16, 18, 19].

### Primary outcome: incidence of postoperative delirium

**Fig. 3:**
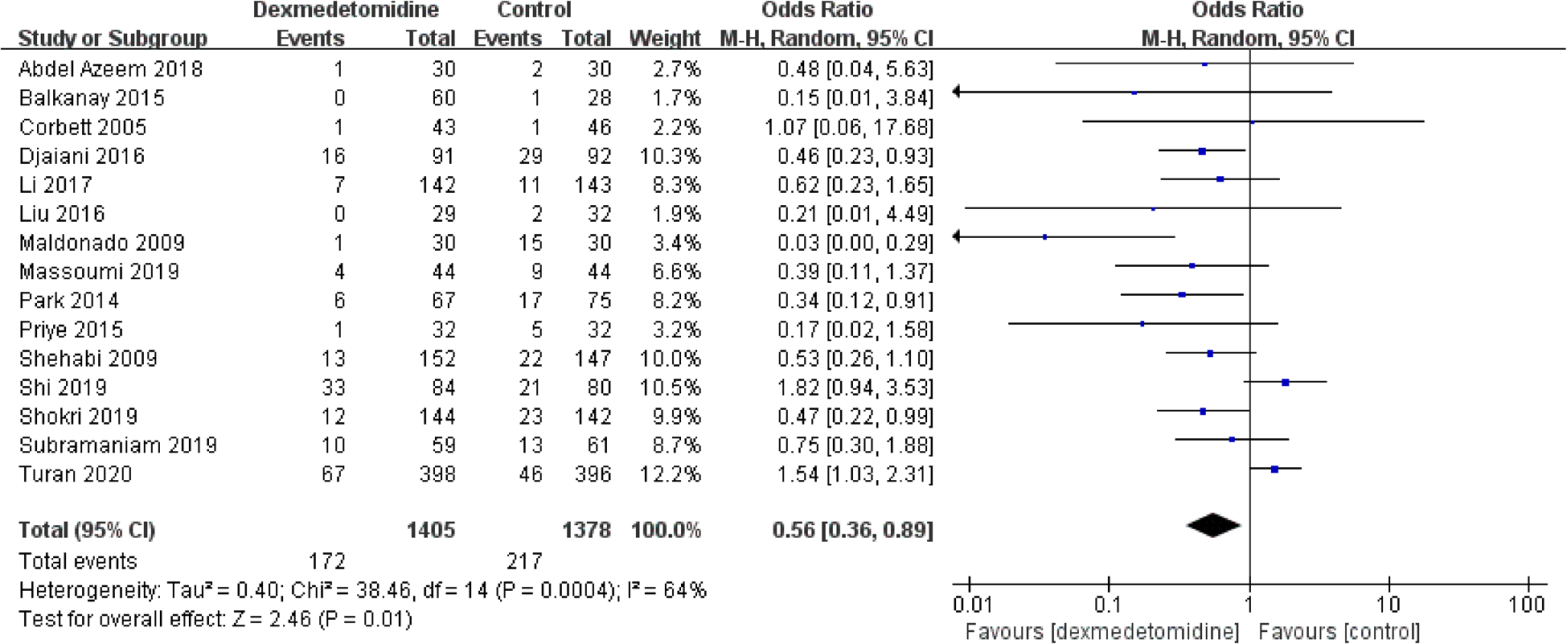
The forest plot of postoperative delirium incidence

**Fig. 4:**
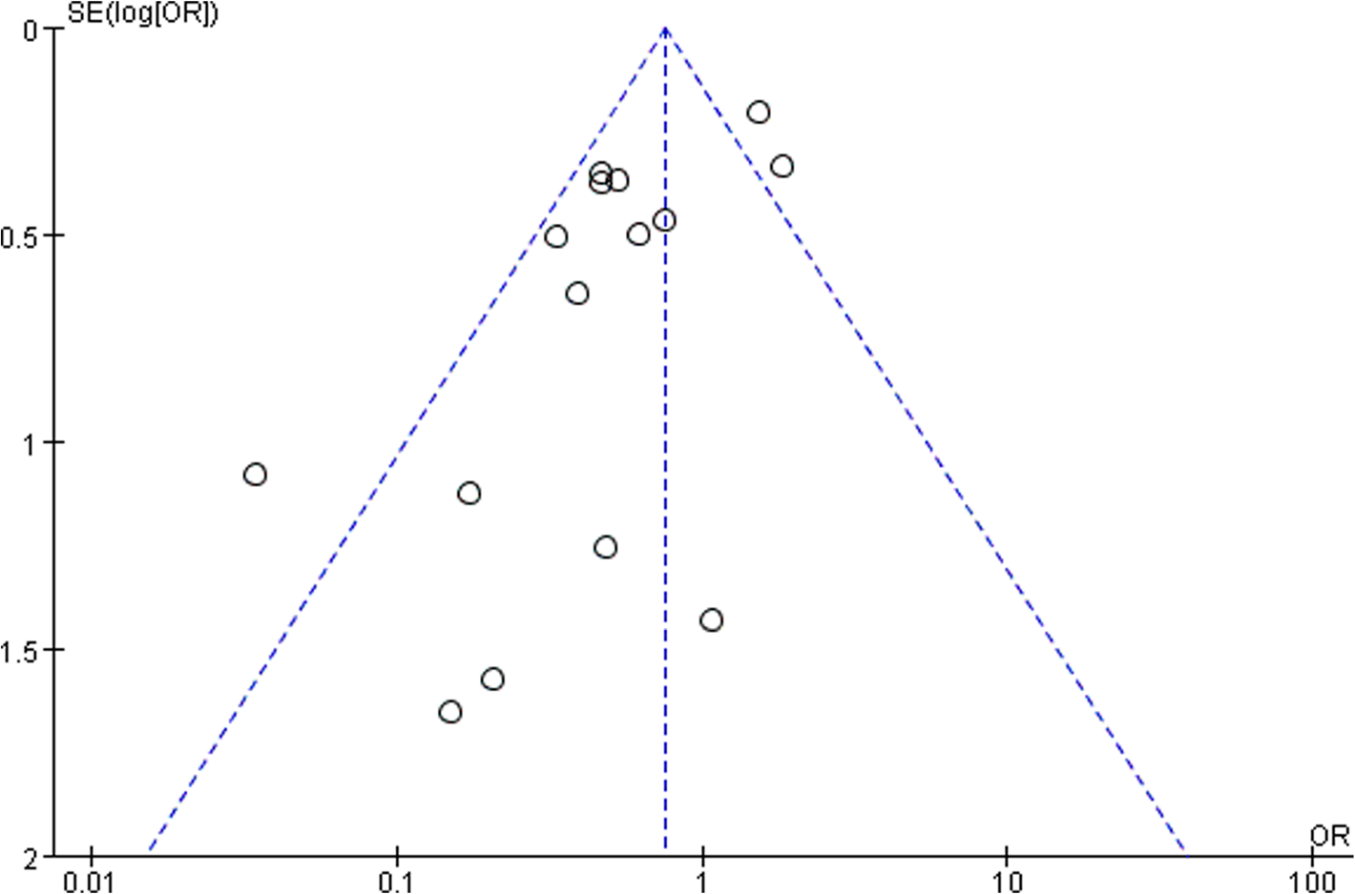
The Funnel plot of postoperative delirium incidence

This meta-analysis revealed that dexmedetomidine can significantly decrease the incidence of POD compared to the controls (OR 0.56, 95% CI 0.36–0.89, *P* = 0.0004, I^2^ = 64%; Fig. 3). Dexmedetomidine reduced the risk of POD by 44%. Funnel plot for the total POD incidence did not suggest the significantly presence of significantly publication bias (Fig. 4).

**Fig. 5:**
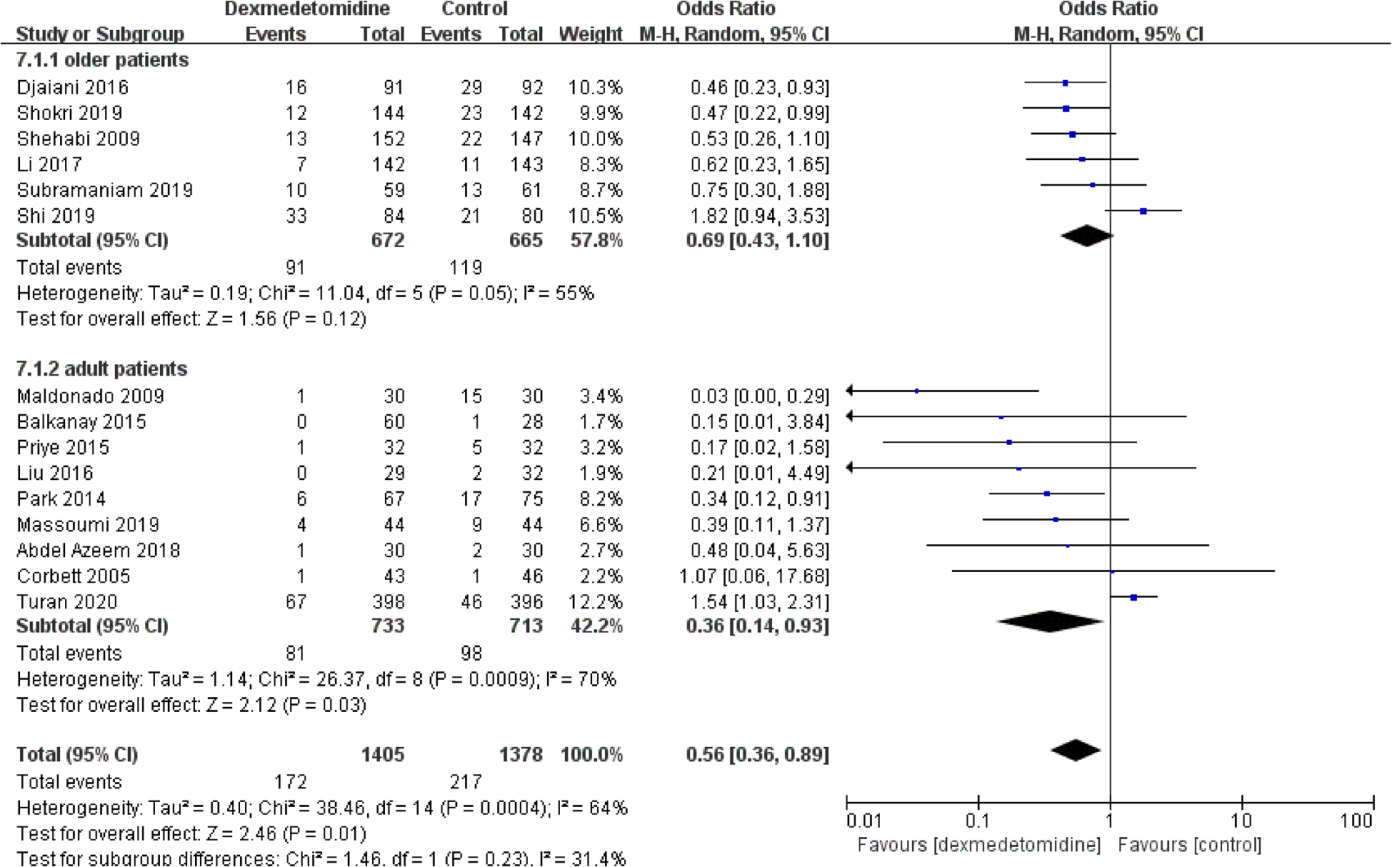
Subgroup analysis of postoperative delirium incidence within different age subgroups

**Fig. 6:**
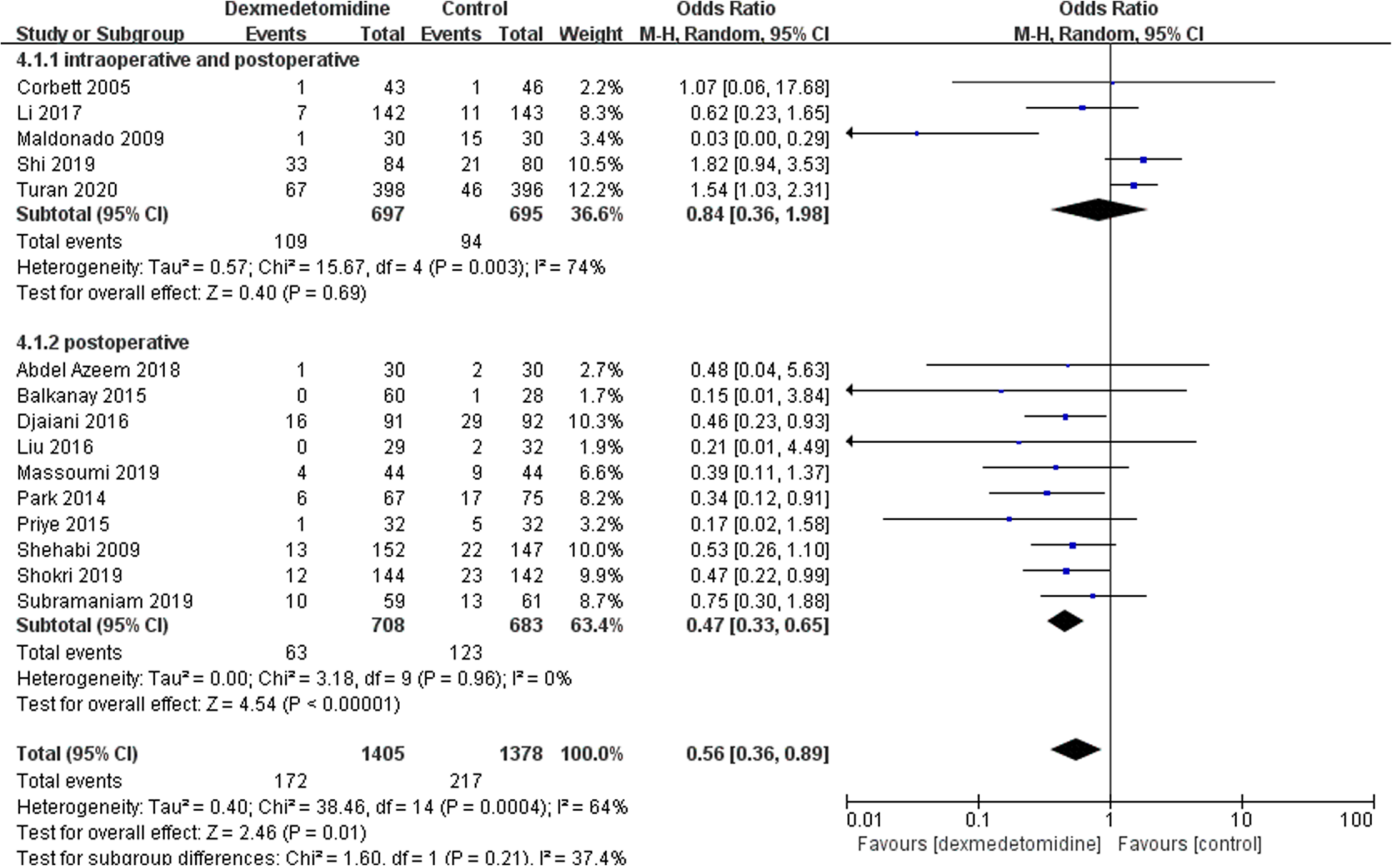
Subgroup analysis of postoperative delirium incidence within different administration time-point

**Fig. 7:**
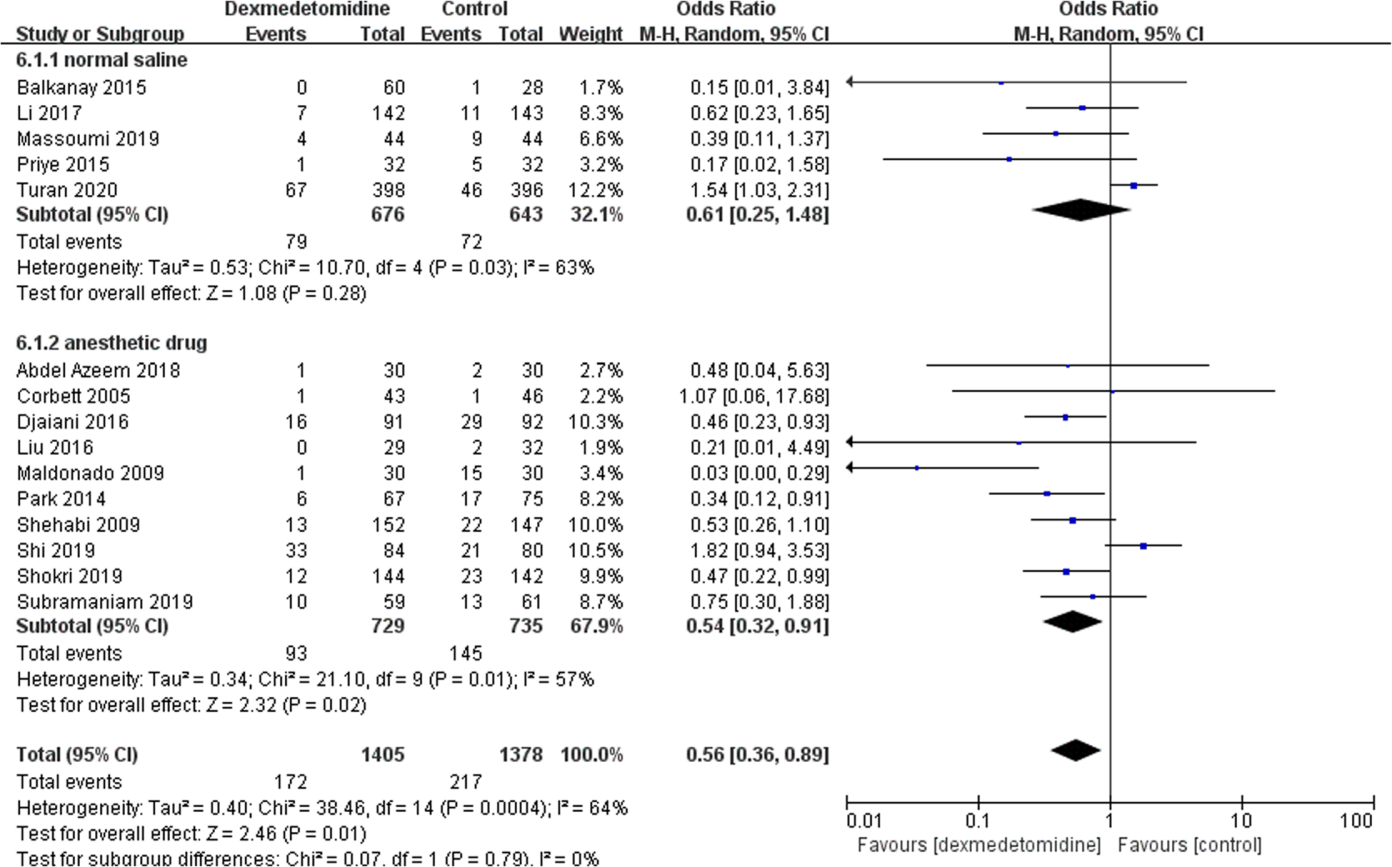
Subgroup analysis of postoperative delirium incidence within different controls

Then we performed a subgroup analysis to find potential sources of heterogeneity and evaluate the risk factors influencing POD. We divide the studies according to potential associated factors, including age (patients older than 65 years or not) (Fig. 5), timing of administration (postoperative only or both intra-and postoperative) (Fig. 6), different controls (normal saline or other anesthetic drugs) (Fig. 7). The subgroup analysis of age showed that dexmedetomidine reduced the POD incidence only in adult population without age restriction, but not in the elderly population (Fig. 5). When the studies were divided according to the timing of administration, it was demonstrated that the incidence of POD was reduced in studies in which dexmedetomidine was administered after surgery, but not in those with intraoperative use (Fig. 6). Interestingly, dexmedetomidine was favored in preventing POD when compared to other sedatives, but not to normal saline (Fig. 7).

### Secondary outcomes: complications

**Fig. 8:**
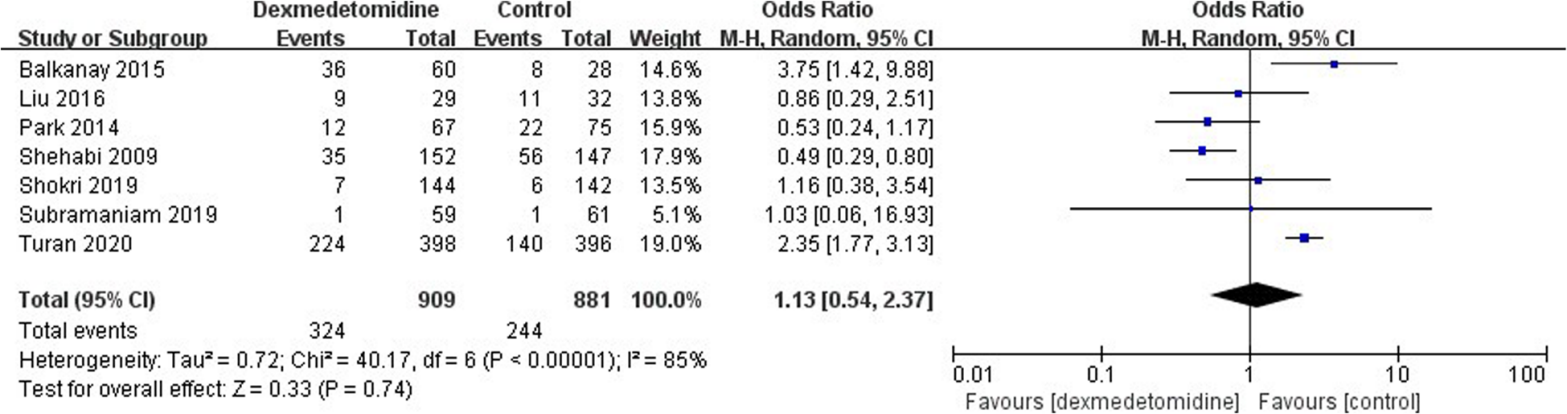
The forest plot of postoperative hypotension incidence

**Fig. 9:**
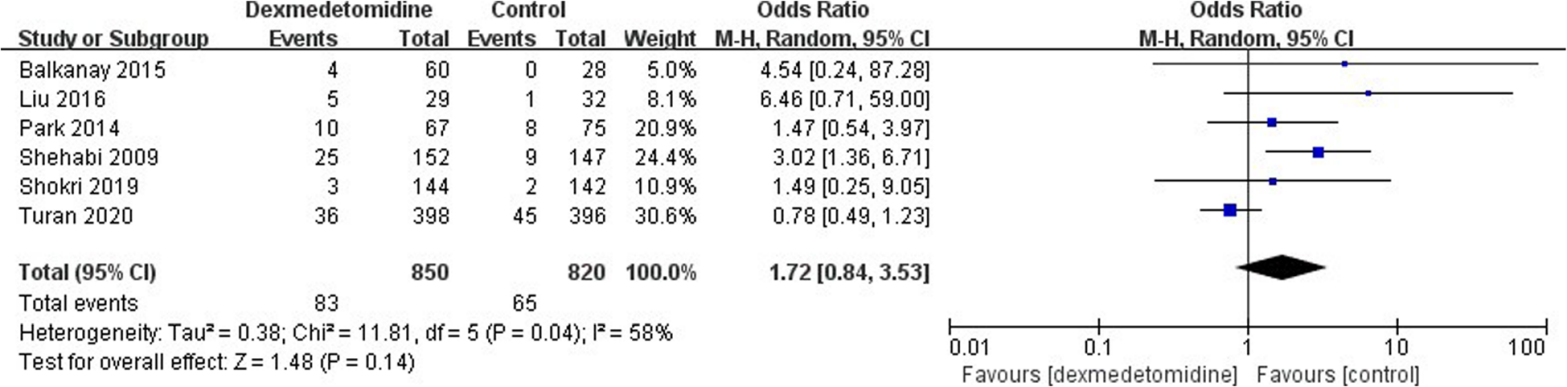
The forest plot of postoperative bradycardia incidence

**Fig. 10:**
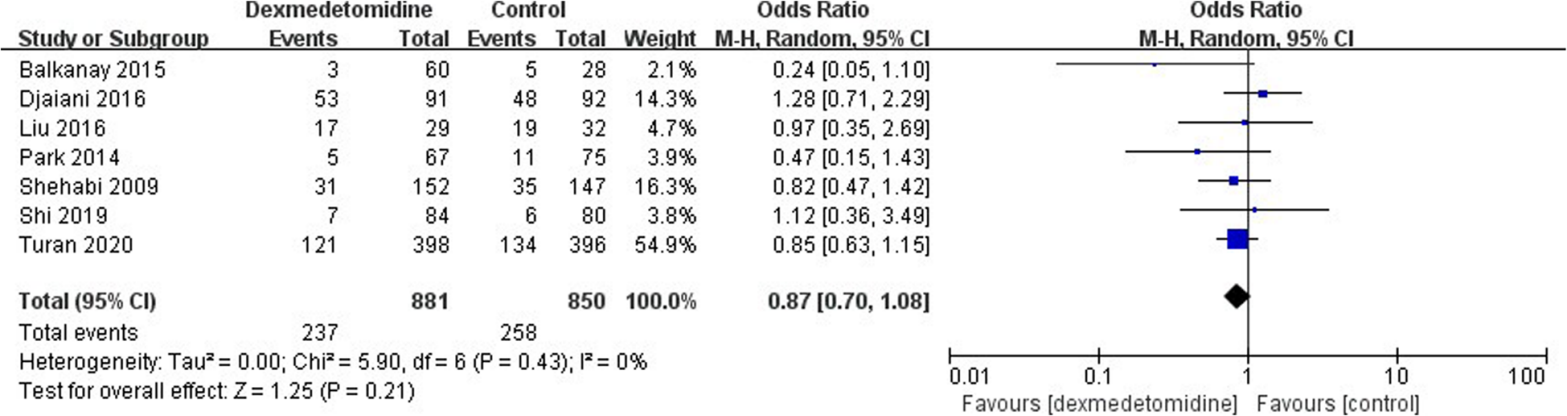
The forest plot of postoperative atrial fibrillation incidence

In the 15 studies included in the meta-analysis,7 studies compared the incidence of postoperative hypotension, but the data was too heterogenous to be pooled though the trend showed a negative result (OR 1.13; 95% CI 0.54–2.37, *P* < 0.00001, I^2^ = 85%) (Fig. 8). The incidence of postoperative bradycardia was compared in 6 studies but no statistical difference was identified (OR 1.72; 95% CI 0.84–3.53, *P* = 0.04, I^2^ = 58%) (Fig. 9). Atrial fibrillation was reported in 7 studies but no significant difference was found either (OR 0.87; 95% CI 0.70–1.08, *P* = 0.43, I^2^ = 0) (Fig. 10).

## Discussion

Our meta-analysis demonstrates that dexmedetomidine can decrease the incidence of POD for adult patients after cardiac surgery, although several recent large-scale trials with negative results were included [9]. The protective effect of dexmedetomidine against POD after cardiac surgery does not seem to be shown in the elderly population. The subgroup analysis also suggests that the ideal time for administering dexmedetomidine may be postoperative period, but not from intraoperative to postoperative period. Most interestingly, dexmedetomidine may reduce the incidence of POD only when compared to other anesthetics, but not to normal saline. The secondary analyses show that there is no significant difference in complications associated with dexmedetomidine infusion, such as bradycardia, hypotension and arial fibrillation. But the number of the included trials may be not large enough to confirm the results of secondary analyses.

The main result of this meta-analysis is not surprising because previous meta-analyses have provided strong evidence for dexmedetomidine in preventing POD after cardiac surgery [7, 8]. But one very resent, large-scale, randomized controlled trial with negative result raised new doubt in the protective effect of dexmedetomidine [9]. The effect of dexmedetomidine against POD might have not been fully clarified. Therefore, we performed the subgroup analyses to investigate the factors influencing the protective of dexmedetomidine against POD after cardiac surgery.

The subgroup analyses seemed to show that dexmedetomidine was not protective against POD in all of the patients undergoing cardiac surgery. Age and the time to start administration were two important factors determining the positive effect of preventing POD. Although the age is a risk factor for POD, dexmedetomidine failed to reduce the incidence of POD after cardiac surgery. However, in non-cardiac surgery, it favors administering dexmedetomidine to prevent POD in an RCT with a relatively large sample size and a previous meta-analysis [12, 25]. The different hemodynamic status between cardiac surgery and non-cardiac surgery might be one of the reasons. Hypotension might be more frequent in cardiac surgery, and especially in the elderly undergoing cardiac surgery [26, 27]. Intraoperative hypotension is a risk factor for POD, and dexmedetomidine may induce intraoperative hypotension, which was not well recorded in most of the clinical trials [26, 27]. Thus, the hypotension induced by dexmedetomidine might be the reason why POD was not protective in the elderly in this meta-analysis [28]. Other researchers also reported that the incidence of possible POD was strongly associated with preoperative exercise capacity in patients undergoing elective cardiac surgery [17]. Compromised exercise capacity might be more common in the elderly, who might be more sensitive to POD after cardiac surgery. This may be why the dexmedetomidine has no protective effect in elderly people. It is interesting to notice that the protective effect was shown in postoperative use of dexmedetomidine, but not the use from intraoperative to postoperative period. Anesthesia depth has been reported to be associated with POD and dexmedetomidine might induce deeper anesthesia depth [26, 28]. But further studies were required to investigate these speculations.

However, the role of arterial blood pressure abnormalities in POD during cardiac surgery remains unclear [26, 27, 29, 30]. Research on the issue has focused on low blood pressure, but with conflicting results [26, 27, 29, 30]. It has been suggested that POD may be associated with high flow and cerebral perfusion during CPB in excess of cerebral metabolic requirements, resulting in excessive brain micro-emboli load, endothelial cell damage, and damage to the blood-brain barrier, leading to cerebral edema, and thereby brain dysfunction [29, 30]. In summary, existing studies suggest an increased risk of POD in a small number of patients with chronic or deep hypotension, but this risk is not statistically significant [31]. Further studies are needed to confirm the effect of blood pressure management on the incidence of POD. In addition, a study of rats showed that blood transfusions increase interleukin-6 levels and lead to neuroinflammation and subsequent cognitive impairment [32].

Sedation with anesthetics or analgesics are common in the intensive care unit (ICU) to allow the patient to remain comfortable, calm, and painless [33]. Sedation and analgesia are required in most of the intensive situations to promote natural sleep, facilitate assisted ventilation, and regulate physiological responses to stress (such as tachycardia and hypertension). The commonly used sedative medications include propofol, morphine, dexmedetomidine, clonidine and benzodiazepines [6]. Compared with propofol, the anti-sympathetic action of dexmedetomidine reduces serum catecholamine, lowers heart rate, increases blood supply to the coronary arteries of the left ventricle by extending diastolic duration and reduces myocardial oxygen consumption [34]. The risk of side effect such as hypotension or vasopressin requirement induced by dexmedetomidine is lower than that induced by other anesthetics or analgesics [34]. Although the secondary analysis suggested that dexmedetomidine did not alter the incidence of postoperative hypotension, but the data was too heterogenous to be pooled. The type of control medication or the frequency of monitoring might also confound the explanation of this result. Thus, we speculated that dexmedetomidine might result in less hemodynamic events and non-physiological sleep, which might be associated with POD. Our data suggested that dexmedetomidine did not reduce the incidence of POD compared with normal saline but it did prevent POD when compared to other anesthetics or analgesics. Therefore, when sedation must be performed, dexmedetomidine might be a better choice than other anesthetics or analgesics.

On the other hand, 5 studies were included in the subgroup analysis of control medication, but according to the sample size of these studies, the study performed by Turan et al. [9] contributed the most weight in this sub-analysis and it might also be one of the important reasons why our meta-analysis demonstrated that dexmedetomidine was not protective against POD after cardiac surgery in comparison to placebo. In the study of Turan et al., anesthesiologists and intensivists were allowed to decrease the dose of dexmedetomidine and other sedatives were allowed to be used when necessary. But the cumulative dose of dexmedetomidine and other sedatives were not quantified, thus we were unable to assess whether patients in the dexmedetomidine group received more sedatives. The increased dose of sedatives might also contribute to the negative result in this study. Therefore, the effect of dexmedetomidine in preventing POD compared to placebo needed to be further investigated in future randomized controlled studies.

There are several potential limitations in our study. Firstly, several recent RCTs with negative results have a significant influence on the heterogeneity of this meta-analysis, especially the one published in 2020 [9] which is outside the funnel plot. Secondly, the present study did not investigate the incidence of organ injury, so it was not possible to demonstrate whether dexmedetomidine was worthy to be used in cardiac surgery from this perspective. Thirdly, in this study, we also did not involve the analyses regarding intraoperative blood pressure, BIS value and other indicators clearly affecting POD, because many studies did not report these data. Therefore, it is not clear whether strict management of hemodynamic status and anesthetic depth would influence the benefit of dexmedetomidine in preventing POD.

## Conclusion

In summary, postoperative infusion of dexmedetomidine can reduce the incidence of POD in adult patients who have undergone cardiac surgery when compared to other anesthetics. But obvious heterogenicity are present in the RCTs published in these studies. Future high-quality, large scale, randomized controlled clinical trials are still needed to verify the protective effects of dexmedetomidine against POD in sub-population of cardiac surgery patients.

## Supplementary Information

**Additional file 1.**

## Data Availability

All data generated or analyzed during this study are included in this published article [and its supplementary information files].

## Abbreviations

DEX: Dexmedetomidine
NS: Normal saline
CAM: Confusion Assessment Method
CAM-ICU: CAM for Intensive Care Unit
RASS: Richmond Agitation Sedation Scale
MMSE: Mini-Mental State Examination
CPB: Cardiopulmonary bypass
CABG: Coronary artery bypass grafting
AF: Atrial fibrillation
